# Unveiling oscillatory nature for sustainable fuel production

**DOI:** 10.1093/nsr/nwae068

**Published:** 2024-02-24

**Authors:** Ning Han, Ye Wang, Bao-Lian Su

**Affiliations:** Department of Materials Engineering, KU Leuven, Belgium; State Key Laboratory of Physical Chemistry of Solid Surfaces, Collaborative Innovation Center of Chemistry for Energy Materials, National Engineering Laboratory for Green Chemical Productions of Alcohols, Ethers and Esters, College of Chemistry and Chemical Engineering, Xiamen University, China; Laboratory of Inorganic Materials Chemistry (CMI), University of Namur, Belgium; State Key Laboratory of Advanced Technology for Materials Synthesis and Processing, Wuhan University of Technology, China

A century ago, in 1923, Franz Fischer and Hans Tropsch introduced the groundbreaking Fischer–Tropsch (FT) synthesis, a heterogeneous catalytic process used to convert synthesis gas (syngas, CO + H_2_) into multi-carbon hydrocarbons [1]. Over the past 100 years, FT synthesis has emerged as a pivotal method for converting non-petroleum resources into exceptionally clean fuels or valuable chemicals via syngas [2–4]. Despite this extensive timeframe, the precise mechanistic steps of this polymerization-type surface reaction remain a topic of ongoing debate. While time-dependent reaction kinetics leading to phenomena like hysteresis and oscillations were demonstrated for CO oxidation over Pt-based catalysts [5], little is currently understood about such kinetic instabilities in reaction networks. In the 1990s, a surface science study on the oscillatory dynamics reaction was also carried out by Prof. Gerhard Ertl [6]. Chemical reactions occurring far from equilibrium on solid surfaces can exhibit characteristic phenomena of nonlinear dynamics, as illustrated by the catalytic oxidation of CO on a platinum(110) single-crystal surface [6]. The temporal variation of the reaction rate may display oscillatory behavior, depending on external parameters such as temperature and partial pressures of the reactants. Similarly, the concentration distributions of adsorbed species on the surface give rise to spatio-temporal patterns, encompassing propagating and standing waves, rotating spirals, as well as irregular and rapidly changing structures referred to as ‘chemical turbulence’.

In the paper entitled ‘The oscillating Fischer-Tropsch reaction’ by the groups of Prof. Yong Wang and Prof. Norbert Kruse, the authors reported periodic rate-and-selectivity oscillations in the FT reaction over a Co/Ce–oxide powder catalyst [7]. After transitioning from reducing conditions in H_2_ to reactive conditions in H_2_/CO, while maintaining a constant flow rate, the catalytically active surface phase-initiated formation. This chemical conditioning consistently preceded the onset of oscillations and was accompanied by delay times of several hundred seconds, contingent upon the specific H_2_/CO ratio and the Co/Ce atomic ratios in Co/Ce–oxide catalysts. The long-term temperature oscillations with periods of approximately 340 seconds and amplitudes of ∼7°C were observed for Co/Ce = 2 and H_2_/CO = 1. Over a 15-hour period, these amplitudes decreased to ∼2°C (Fig. 1a and b). The changes in amplitude occurred gradually over ∼1 hour, rather than abruptly. The origin of this amplitude damping could be linked to alterations in the surface composition of the catalyst due to the adsorption of contaminating species. The oscillating temperatures reflected periodic shifts in the exothermic nature of the reaction, which was further confirmed by the harmonic nature of the high-amplitude oscillations. Efficient thermal coupling between distant catalyst particles was essential to ensure the seemingly synchronized reaction behavior across the catalyst bed. It was directly confirmed that the swift heat exchange between catalyst particles and the gas phase, facilitated by the high thermal conductivity of He, was disrupted upon replacement with Ar, indicating the importance of Ar for the efficient thermal coupling between catalyst particles during FT.

**Figure 1.**
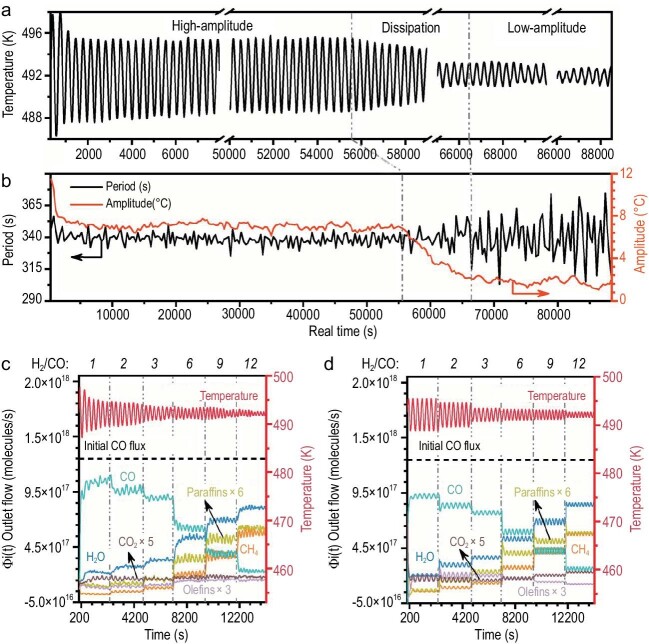
(a) Self-sustained long-term oscillations of temperature over the Co/Ce–oxide catalyst. (b) Oscillating temperatures with periods. (c) Experiment and (d) simulation of the oscillation behaviors. Reprinted with permission from [7].

Figure 1c and d compared the results of forced temperature oscillations with naturally occurring nonisothermal oscillations observed experimentally. The temperature variations were primarily attributed to Arrhenius factors associated with C−O bond breaking. The time-averaged outlet flows of reactants and products deviated by ∼20% (usually even less) from each other across the entire range of H_2_/CO ratios. These promising findings elucidate the occurrence of rate and selectivity oscillations in the classical FT reaction over cobalt/ceria catalysts. It is intriguing to explore the possibility of generating self-sustained oscillatory behavior based on a thermokinetic feedback mechanism, eliminating the need for externally induced temperature oscillations. Overall, the concordance between experimental and theoretical oscillations has been thoroughly demonstrated across a broad spectrum of reactant pressure ratios. This alignment strongly supports the CO insertion reaction mechanism, which was developed by the same research group using chemical transient kinetics (CTK) [8,9], for the formation of alcohols and ketones. A similar behavior was also observed in toluene total oxidation over Pd-Au catalysts [10].

This study has the potential to inspire more in-depth research aimed at identifying the precise parameters necessary to initiate and investigate kinetic instabilities in complex catalytic reactions, such as the FT reaction. The results from this research further support the idea of adopting a dynamic catalyst design strategy, which would enhance the reaction rate and selectivity by harnessing oscillatory reaction states.

## References

[1] Fischer F, Tropsch H. Brennst Chem 1923; 4: 276–85.

[2] Torres Galvis HM, Bitter JH, Khare CB et al. Science 2012; 335: 835–8. 10.1126/science.1215614 22344440

[3] Navarro V, Spronsen MAV, Frenken JWM. Nat Chem 2016; 8: 929–34. 10.1038/nchem.2613 27657868

[4] Zheng Q, Williams J, Thiel LRV et al. Nat Catal 2023; 6: 185–95. 10.1038/s41929-023-00913-8

[5] Beusch H, Fieguth P, Wicke E. Chemieingenieurtechnik 1972; 44: 445–51.

[6] Ertl G . Science 1991; 254: 1750–5. 10.1126/science.254.5039.1750 17829239

[7] Zhang R, Wang Y, Gaspard P et al. Science 2023; 382: 99–103. 10.1126/science.adh8463 37797023

[8] Bundhoo A, Schweicher J, Frennet A et al. J Phys Chem C 2009; 113: 10731–9. 10.1021/jp902647z

[9] Schweicher J, Bundhoo A, Kruse N. J Am Chem Soc 2012; 134: 16135–8. 10.1021/ja3068484 22992066

[10] Barakat T, Rooke JC, Chlala D et al. Catalysts 2018; 8: 574. 10.3390/catal8120574

